# How Learning Motivation Influences Feedback Experience and Preference in Chinese University EFL Students

**DOI:** 10.3389/fpsyg.2020.00496

**Published:** 2020-03-20

**Authors:** Zhengdong Gan

**Affiliations:** Faculty of Education, University of Macau, Macau, China

**Keywords:** feedback experience, feedback preference, English learning motivation, structural equation modeling, quantitative research

## Abstract

Drawing on the argument that students’ different learning behaviors, including their perceptions of and engagement with feedback, could have roots in learners’ fundamental motivational characteristics, this study examines how different second language learning motivational variables may predict university EFL (English as a foreign language) students’ feedback experience and preference. Data were collected from EFL students from three universities in an Asian region (*N* = 409) through three self-report questionnaires. Results of structural equation modeling (SEM) revealed that different components of the second language learning motivational construct appear to display differential associations with EFL students’ feedback experience and preference. In particular, this study brought to light the crucial role of *attitudes to classroom English learning* and *intended learning effort* as essential mediating motivational variables in predicting how EFL students conceive of and act on feedback. The findings of this study also provide significant insights into a complex and dynamic view of how student preference for different types of feedback actually works in the feedback process. The authors conclude by arguing that EFL teachers need to shoulder the burden of making the EFL classroom a supportive environment that promotes a positive self-concept and self-confidence as the first step toward stimulating students’ active feedback use, and that conditions need to be created to allow for connection of students’ preference for learning process-oriented feedback to action to maximize the pivotal role that students play in the classroom and learning process.

## Introduction

For several decades, researchers in educational psychology and assessment have considered feedback as an important component in student learning and developmental progress. Feedback has been recognized among researchers to be a potential powerful tool for improving students’ learning and reducing the gap between where students are and where they need to be as feedback enables students to evaluate their own work and thereby enhancing their self-regulation (Leung et al., 2018; Jørgensen, 2019). In second language acquisition, feedback has been depicted as being essential to the process of target language learning and skill acquisition as it plays an essential role in maintaining interaction between lecturers and students, or between peer students (Hyland and Hyland, 2006). Since opportunities for interaction are usually limited in an EFL context, such feedback-facilitated interaction tends to be viewed by second language researchers as a major factor contributing to not only students’ target language development but also extending of their autonomous learning activities (Hyland, 2003). Second language feedback research, however, has focused on teachers’ feedback practices dominated by product-based and error-focused orientations that treat students as passive feedback recipients (Papi et al., 2019). This type of feedback research typically analyzed written teacher comments (i.e., corrective feedback) to students and examined uptake of such feedback on the part of students, but failed to explore how feedback comments are actually received and acted upon (Ajjawi and Boud, 2017). As Ajjawi and Boud (2017) rightly point out, even if some studies reported on the effects of different types of feedback on student learning performance, these studies usually did not investigate the processes involved and how these effects were achieved. Consequently, although there has been an increasingly large body of research on corrective feedback in second language acquisition, the issue of corrective feedback remains a controversial topic and far from being completely understood (Waller and Papi, 2017). According to Papi et al. (2019), a major drawback in the current second language feedback research is the lack of sufficient attention to the role of individuals in the learning process, and students’ feedback experience and motivated involvement in the feedback process has virtually not been researched. After all, differences in students’ learning behaviors, including their perceptions of and engagement with feedback, could have roots in learners’ fundamental motivational characteristics (Papi and Teimouri, 2014). Drawing on recent feedback research in higher education to re-conceptualize feedback practices and re-cast students as active agents in the feedback processes (Ajjawi and Boud, 2017; Carless and Boud, 2018), the present study intends to fill the gap in the second language feedback literature by investigating how different second language learning motivational variables interact to influence EFL students’ feedback experience and preferences in an Asian EFL setting. It is believed that feedback research in second language acquisition can benefit from drawing on advances in feedback in higher education.

### Theorizing Feedback

Despite the importance ascribed to the role of feedback in students’ construction of knowledge and skills, there has been a range of different views about the concept and practice of feedback in the literature. In keeping with learning theory paradigms of behaviorism and information processing, feedback used to and may still be regarded as a strong external stimulus providing positive or negative reinforcement to behavior, where feedback is conceptualized as an issue of “knowledge of results” or “correction of errors,” and where the role of feedback is to “put things right” by taking a corrective action (Gibbs and Simpson, 2004). This cognitivist, corrective view of feedback as an end product from the teacher to the learner views feedback as a unidirectional transmission of knowledge, which frequently arises from the dominant structural constraint of written comments on end of course assignments (Yang and Carless, 2013). In fact, this one-way communication of written feedback comments represents what Carless (2015) describes as the “old paradigm” of feedback practice, which characterizes students as passive recipients of feedback information. The main purpose of this type of feedback is to confirm or change a student’s knowledge as represented by answers to test or assignment questions (Butler and Winne, 1995, p. 246). It is usually not given during learning activities but is given by a tutor after a task has been completed or a test of achievement has been administered (Butler and Winne, 1995). Indeed, many institutions appear to favor the transmission model of feedback, intentionally or unintentionally positioning students as mechanical receivers of information about their academic work (Winstone and Boud, 2019). Reinforcing this position is a conviction that feedback practices are necessarily situated within the social and cultural contexts as the processes of assessing and informing progressions in students’ learning performance are ecologically rational representations of the traditions and values that teachers experience within these contexts. In an EFL context such as Asia characterized by a highly competitive examination-driven education system, teachers have traditionally been seen as subject experts in the classroom and may be reluctant to rescind their traditional authority. This may be one potential explanation for why feedback as information transmission or “telling” has been dominant particularly in the Asian teaching context.

In recent years, however, informed by the socio-constructivist view of learning, researchers tend to reframe feedback as an active and collaborative process that positions the learner as an agent in bringing about improvement in their learning (Carless and Boud, 2018), and the spotlight has thus shifted from viewing feedback as teachers informing students about strengths and weaknesses of their work to recognizing feedback as an iterative process through which students make sense of information from various sources and use it to enhance their work or learning strategies. This appears to be best reflected in Boud and Molloy’s (2013) definition that “feedback is a process whereby learners obtain information about their work in order to appreciate similarities and differences between the appropriate standards for any given work, and the qualities of the work itself, in order to generate improved work” (p. 205). This emphasis on feedback process represents what Carless (2015) termed as the “new paradigm” of feedback practice, and represents “a very different way of thinking, with an expectation of students” active engagement with feedback information they receive, and a focus on the resulting improvements in subsequent tasks” (Winstone and Boud, 2019, p. 412). Indeed, the reconceptualization of feedback has given rise to the call for the dominant transmission model of feedback characterized by teacher-controlled delivery to move away toward a focus on student engagement with feedback in line with a socio-constructivist view of learning (Price et al., 2011). In essence, the most important part of this socio-constructivist view of feedback is that feedback requires active student roles in making decisions about the feedback received in order to improve learning (Boud and Molloy, 2013), feedback enhances development of the capacity to regulate students’ learning (Hattie and Timperley, 2007), and feedback needs to address future-oriented longitudinal development (Carless and Boud, 2018).

In the area of second language acquisition, a large body of feedback research following the cognitivist information transmission approach and examining different dimensions of feedback (e.g., feedback types, feedback frequency, and feedback timing, etc.) in relation to their effects on learning (e.g., Saito and Lyster, 2012; Lyster et al., 2013; Bitchener and Storch, 2016) has so far yielded many conflicting findings (Shute, 2008). According to Shute, one potential explanation for the lack of consistent pattern of results in these studies is a function of individual differences among motivational prerequisites (e.g., intrinsic motivation, beliefs, and academic self-efficacy). In second language acquisition, there were also studies that took a socially grounded approach and investigated the effectiveness of scaffolded and dialogically negotiated feedback (e.g., Nassaji, 2015). While these studies can enable insight into previously undocumented dimensions of feedback, such as feedback as a dialogically co-constructed practice (Majlesi, 2018), the main purpose of these studies was to explore “the reasons for learners’ receptiveness to corrective feedback, or lack thereof, rather than their proactive involvement in the learning pursuit” (Papi et al., 2019, p. 206). As Papi et al. noted, feedback in these studies has been treated mainly as a teaching resource rather than a learning resource, and students’ motivated involvement in the feedback process has largely remained unexplored.

### Self-Determination Theory

One of the psychological theories of motivation that integrate motivation and cognition is self-determination theory (SDT), proposed by Deci and Ryan (2000). From a SDT perspective, individual motivation is defined as the degree of autonomy that individuals display during learning activity, and it falls into two major motivational orientations: (1) self-determined forms of intrinsic motivation; and (2) controlled forms of extrinsic motivation. According to Deci and Ryan, intrinsic motivation denotes that a learner engages in an activity for its own sake in order to experience the pleasure and satisfaction, and it is characterized by interest, enjoyment, and intense involvement. In other words, intrinsically motivated students perform tasks because they find them enjoyable, interesting and that participation is its own reward, which in turn should further motivate their investment of task-directed effort (Dysvik and Kuvaas, 2013). Significantly, intrinsically motivated students not only seek feedback from external sources such as peer classmates’ contributions in collaborative groups, or teachers’ remarks on work done in class, but also develop idiosyncratic cognitive routines for creating internal feedback while they are engaged with academic tasks (Butler and Winne, 1995). In addition, intrinsic motivation has been shown to be associated with the use of strategies of higher level thinking and optimal learning outcomes (Thomas and Oldfather, 1997). Unlike intrinsic motivation, extrinsic motivation focuses more on the consequences to which the task leads than on the task itself (Gagne and Deci, 2005; Dysvik and Kuvaas, 2013). In other words, learners behave to obtain a desired consequence such as tangible rewards, and the resulting behavior is usually instrumental rather than being done as a source of spontaneous enjoyment and satisfaction (Deci and Ryan, 2000). This suggests that the task is a means to an end, but the requirement to perform the task is imposed on the learner and the learner may not feel like doing the task (Cheng and Fox, 2017). While research has revealed generally mixed results concerning the relationships between extrinsic motivation and use of language learning strategies and learning outcomes, associations between extrinsic and intrinsic motivation have been shown to be positive and strong (Gonzales, 2011).

### The L2 Motivational Self System and the Related Research

Motivation is widely believed to be an important factor contributing to second or foreign language learning outcomes and has been the subject of intensive research in the past two decades (Lamb and Arisandy, 2019). One of the leading models of learner motivation in the literature is Gardner’s (2010) socio-educational model of L2 motivation. Gardner described motivation as “the combination of effort plus desire to achieve the goal of learning the language plus favorable attitudes toward learning the language” (Gardner, 1985, p. 10). A central concept in language learning motivation in Gardner’s (2010) socio-educational model is the notion of “integrativeness” – a desire to learn a language in order to “come closer to the other language community” (Gardner, 2001, p. 5). According to this model of learner motivation, integratively motivated language learners demonstrate not only favorable attitudes toward the target language community and the people who speak the target language, but also positive attitudes toward the learning situation and exhibited aspects of motivated behavior such as effort, an expressed desire and enjoyment in the process of learning (Lamb, 2004).

More recently, building on personality psychology research on possible selves (Higgins, 1987) and the motivational constructs by Noels (2003) and Ushioda (2001), Dörnyei (2005, 2009) proposed of a new conceptualization of L2 motivation, the L2 Motivational Self System. An essential dimension of the L2 Motivational Self System is the ideal L2 self, which, according to Dörnyei, denotes the representation of the attributes that someone would ideally like to possess. Put simply, ideal L2 self refers to an image of a perfect future self that synthesizes every desirable characteristic that the individual wishes to possess, such as prosperity, happiness, success, achievement, and – in the case of L2 learners – the target language competence (Moskovsky et al., 2016). Another self-guide as a theoretical construct in the L2 Motivational Self System is the ought-to self, which refers to the attributes that one believes one ought to possess (i.e., a representation of someone else’s sense of duty, obligations or responsibilities) (Dörnyei, 2009). The source of the ought-to self can be understood as being located outside of the learner, denoting a reflection of what others expect to see in this student (Moskovsky et al., 2016). That said, challenging the then-dominant view of integrative motivation for language learning, Dörnyei reasoned that if target language competence is an essential component of learners’ ideal or ought-to self, this will be conceived as a powerful driving force for learners learning the target language because of the learners’ psychological desire to reduce the discrepancy between their current and possible future selves. A third dimension of Dörnyei’s L2 Motivational Self System is the L2 learning experience, which describes situation-specific motives associated with the immediate learning environment and experience (Dörnyei, 2009). The difference between L2 learning experience as a motivational construct and ideal L2 self and ought-to self as two self guides is that L2 learning experience deals with aspects of the learning situation (e.g., teacher-student relationship, learning materials, teaching approaches, classroom environment, and learner beliefs about language learning, and learner capacity foe self-regulation) whereas the two self guides have a strictly future orientation (Moskovsky et al., 2016).

Taguchi et al. (2009) focused on the relationship between the three dimensions of Dörnyei’s new motivational theory illustrated above and learners’ intended learning efforts, and provided support for the importance of the ideal L2 self as a key motivational element of Dörnyei’s theory, although the ought-to L2 self did not emerge as a variable distinct from instrumental orientation in other studies (e.g., Csizér and Kormos, 2009). However, most recently, these studies have been criticized for their methodological limitations (Oga-Baldwin, 2019). In Dörnyei’s model, intended effort (a proxy for motivation) is in fact treated as an outcome variable. According to Oga-Baldwin (2019), intent may not be used as an appropriate means to indicate what learners do. It is therefore suggested that a better approach would be to use engagement to measure the mediating, long-term effects of Dörnyei’s or any other L2 motivation specific model. As Oga-Baldwin states, engagement differs significantly from motivation and intended effort, and phenomena such as “students paying attention in class, interacting with their teacher and classmates, and thinking about learning material” (Oga-Baldwin, 2019, p. 4) are generally grouped under a single umbrella concept of engagement. As such, student feedback engagement can be seen an example of this type of engagement.

In the literature of L2 feedback, learner individual differences have been acknowledged to play a role in determining student engagement or disengagement with feedback, and that the extent to which students are motivated in their learning shapes the extent to which they may choose to accept and proactively act on feedback productively (Hyland, 2003). Nevertheless, empirically, little is known about the interplay between second L2 motivational factors and student feedback engagement (Ellis, 2010). We concur with Carless and Boud (2018) who emphasizes that unless students are motivated to act on feedback, they may have limited potential to occupy a central role in the feedback process. Clearly, to promote feedback effectiveness in the second language education setting, it is important to examine the associations between second language learning motivation and students’ feedback perceptions and feedback action. Furthermore, exploring the associations between L2 motivational variables and student feedback experience will provide evidence of external validity to Dörnyei’s L2 Motivational Self System discussed above. This study, therefore, intends to fill the research gap by examining how language learning motivation in an EFL context may predict EFL students’ feedback experience and preference in order to promote feedback effectiveness. In other words, in the present study, second language learning motivational variables are explored as possible antecedents of students’ feedback preference and feedback experience.

### Research Questions

Specifically, informed by recent new visions in feedback research discussed above, the overarching research question this study addresses is:

What are the relationships between English learning motivational factors and EFL students’ feedback experience and preference?

Two sub-research questions are also specified below:

1. What may characterize Chinese university EFL students’ feedback experience and preference, and English learning motivation?
2. Are there differences in feedback experience and preference between first-year, second-year, and third-year students?

## Materials and Methods

### Participants

In total, 409 English-major students from four Chinese universities participated in the study. Among these participants, 134 students were in their first year; 91 were in their second year; 175 were in their third year; nine did not report which year they were in. Year-4 students did not participate in this study as they were preoccupied with teaching practice. There were 45 male and 362 female students. Two did not report their gender status. The age of the participants ranged from 17 to 23 years with *M*_age_ = 19.64 years, SD_age_ = 1.13 years. In the course of recruiting these participants, approval was obtained from the Research Ethics Committee of the author’s university prior to data collection. The participants were informed that their participation in this study was voluntary, and that they could withdraw from the research at any time if they wanted.

### Instruments

Three questionnaires were specifically constructed for the current study, i.e., the EFL Student Feedback Experience Questionnaire (SFEQ), the EFL Student Feedback Preference Questionnaire (SFPQ), and the EFL Student Learning Motivation Questionnaire (SLMQ). The items in these three questionnaires originated from three major sources: (1) constructs related to classroom feedback practices identified by Hyland (2003); Gibbs and Simpson (2004), Hattie and Timperley (2007), and Zhang and Rahimi (2014), and constructs of motivation identified by Dörnyei (2005, 2009); (2) interviews with some tertiary-level students about the assessment practices they experienced in their classroom, and motivational factors they perceived as a driving force for their English learning; (3) some existing feedback practice and language motivation questionnaires (e.g., Gibbs and Simpson, 2004; Taguchi et al., 2009; Papi, 2010; Crommelinck and Anseel, 2013; Kormos and Csizér, 2014; Zhang and Rahimi, 2014). These processes resulted in an initial item pool of items for each of the questionnaires used in this study. These items were then subjected to a review and examination by professional academics in the fields of classroom feedback practices and language motivation to examine the face and content validity of the items generated. An item in each questionnaire was retained only if both the two professional academics agreed that the item was appropriate to be used to measure feedback practices and English learning motivation in the Chinese higher education. As a result of this validation process, 12 items were retained in the SFEQ: (1) *Quantity and quality of feedback* (6 items); (2) *Feedback use* (6 items). 17 items were retained in SFPQ: (1) *Preference for learning process-oriented feedback* (6 items); (2) *Preference for student self-feedback* (5 items); 3) *Preference for teacher evaluative feedback* (6 items). 15 items were retained in the SLMQ: (1) *Ideal L2 self* (5 items); (2) *Attitudes to classroom English learning* measured (3 items); (3) *Intended learning effort* (7 items). It needs to be pointed out that according to Kormos and Csizér (2014), an inherent difficulty in using surveys in quantitative research is that one needs to restrict and simplify the number of factors that can be analyzed in a single study. We therefore selected three motivational variables that proved most important in influencing language learning processes in previous research in the EFL context (Csizér and Kormos, 2009; Papi, 2010).

As the first language of the participants in this study was Chinese, the three English questionnaires were translated into Chinese by the author, and then, to further ensure the validity of the questionnaires, the Chinese versions were independently translated back into English by two native Chinese-speaking colleagues to see whether anything could be misinterpreted. Finally, a class of twenty EFL students were invited to fill in the Chinese versions of the questionnaires and to comment on the questions. Based on their input, some slight changes were made to the wording of a number of items. This process helped to ensure that the questions in the questionnaires matched the purpose of this study.

In keeping with previous studies, the SFEQ used a 6-point rating scale from1 (strongly disagree) to 6 (strongly agree); the SFPQ used a 6-point rating scale, ranging from 1 (strongly dislike) to 6 (strongly like); the SLMQ also used a 6-point rating scale, ranging from 1 (strongly disagree) to 6 (strongly agree). The following list contains the name of each variable in each of the three questionnaires together with its definition and an illustrative example.

(1) *Quantity and quality of feedback*: students’ perception of the overall quantity and quality of the feedback they received from their teacher. Example: Once I have read the feedback I understand why I got the mark I did.
(2) *Feedback use*: acting on the feedback provided by the teacher. Example: I look over previous feedback when preparing a new assignment.
(3) *Preference for learning process-oriented feedback:* feedback aimed at strategies underpinning particular learning tasks. Example: Teacher provides information on how to improve my spoken English.
(4) *Preference for student self-feedback*: feedback students give to themselves, or feedback given to one another among students themselves. Example: My classmates give me comments about my assignment.
(5) *Preference for teacher evaluative feedback*: feedback about whether work was correct or incorrect. Example: Teacher corrects students’ vocabulary, grammar, and pronunciation errors.
(6) *Intended learning effort:* students’ efforts in learning English. Example: I am willing to work hard at learning English.
(7) *Ideal L2 self:* students’ views of themselves as successful second language speakers. Example: I like to think of myself as someone who will be able to speak English.
(8) *Attitudes to classroom English learning:* students’ perceptions about their classroom English learning experience. Example: I enjoy the activities of our English class much more than those of my other classes.

### Data Analytical Procedure

The data was subjected to confirmatory factor analysis (CFA) using the maximum likelihood (ML) estimation to examine how well the empirical data fit the predetermined factor structure in each of the questionnaires used in this study. Data analysis thus started with testing of the measurement models for the latent variables using CFAs. This was followed by testing the full structural model using SEM. As suggested by Kline (2011), CFA is a statistical measure frequently used to test a theoretical model and a tighter specification of multiple hierarchies by utilizing the factor, correlation, and covariance patterns, and residual or error values within a data matrix, which is an essential step before integrating them into a full structural model (Hair et al., 2006).

The reliability of each instrument used in this study was assessed by examining the internal consistency reliability. Several criteria were used to assess the adequacy of the measurement models and the ensuing full structural model. First, in line with Kline (2011), a chi-square statistic, along with its degrees of freedom (df) and associated *p-*value, was reported. In addition to the chi-square statistic, the following fit indices were chosen to evaluate the model fitness (Byrne, 1998; Hooper et al., 2008): the comparative fit index (CFI; >0.90 indicates good fit), the Tucker-Lewis index (TLI; >0.90 indicates good fit), and the standardized root mean square residual (SRMR; <0.08 indicates acceptable good fit), the root mean square error of approximation (RMSEA; <0.08 indicates acceptable good fit). Furthermore, error terms were correlated when a relatively high and positive correlation was found between the residuals of questionnaire items.

The questionnaire data were analyzed using SPSS 24.0. Descriptive statistics (e.g., mean and standard deviation) in relation to the students’ feedback experience, feedback preference and English learning motivation were reported. MANOVA was conducted to examine whether there were differences in feedback experience and preference between first-year, second-year, and third-year students. To reduce the chance of a Type 1 error, a Bonferroni adjustment was applied. In the case of having five dependent variables, alpha level is set at 0.05/5, i.e., 0.01.

Finally, a one-way repeated measures ANOVA was conducted to compare means for the students’ three types of feedback preference (i.e., preference for process-oriented feedback, preference for self-feedback, and preference for teacher evaluative feedback).

## Results

### Initial Analysis of the Feedback Experience Questionnaire

**FIGURE 1:**
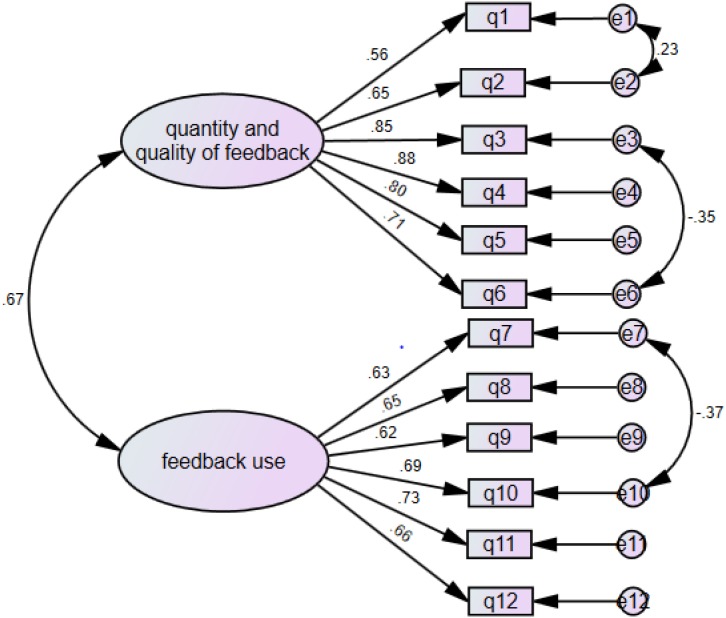
The first-order model of feedback experience.

Confirmatory factor analysis was conducted to determine whether the SFEQ responses fit the pre-determined two-factor feedback experience model. After removing one item due to weak factor loading (with loadings less than 0.4), CFA for the measurement model with the remaining feedback experience items (Figure 1) resulted in satisfying model fits with χ^2^ = 177.23, df = 50; CFI = 0.94; TLI = 0.93; SRMR = 0.048; RMSEA = 0.079. The Cronbach’s α for the two subscales of this two-factor feedback experience model were 0.877 (*quantity and quality of feedback*, 6 items) and 0.812 (*feedback use*, 6 items) respectively, indicating satisfactory levels of internal consistency. Given these CFA results and the Cronbach’s alpha scores, we concluded that the organization of the EFL student feedback experience scale into two distinct dimensions is reliable and valid in keeping with acceptable indices of reliability and validity.

### Initial Analysis of the Feedback Preference Questionnaire

**FIGURE 2:**
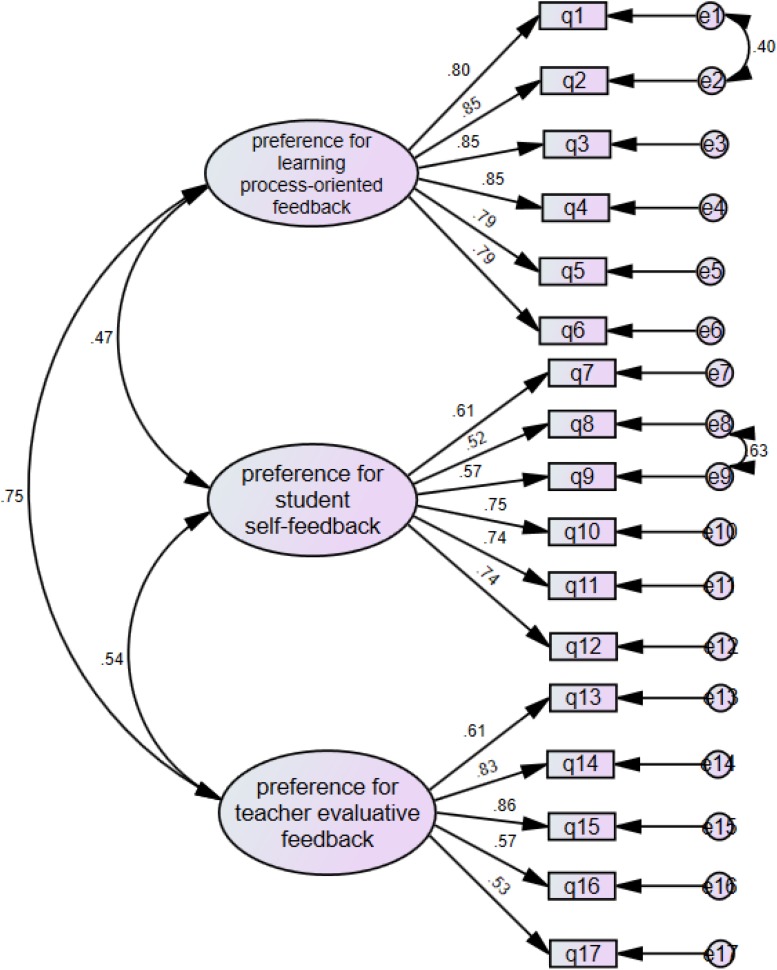
The first-order model of feedback preference.

Confirmatory factor analysis was also conducted to determine whether the SFPQ responses fit the pre-determined three-factor feedback preference model. Satisfactory model fits were found with χ^2^ = 401.706; df = 114; CFI = 0.93; TLI = 0.92; SRMR = 0.052; RMSEA = 0.078. The Cronbach’s α for the three subscales of this three-factor feedback preference model (Figure 2) were 0.928 (*preference for learning process-oriented feedback*, 6 items), 0.834 (*preference for student self-feedback*, 6 items), and 0.818 (*preference for teacher evaluative feedback*, 5 items), respectively, indicating very satisfactory levels of internal consistency. These CFA results and the Cronbach’s alpha scores thus suggest that the organization of the EFL student feedback preference scale into three distinct dimensions is both reliable and valid in accordance with good indices of reliability and validity.

### Initial Analysis of the English Learning Motivation Questionnaire

**FIGURE 3:**
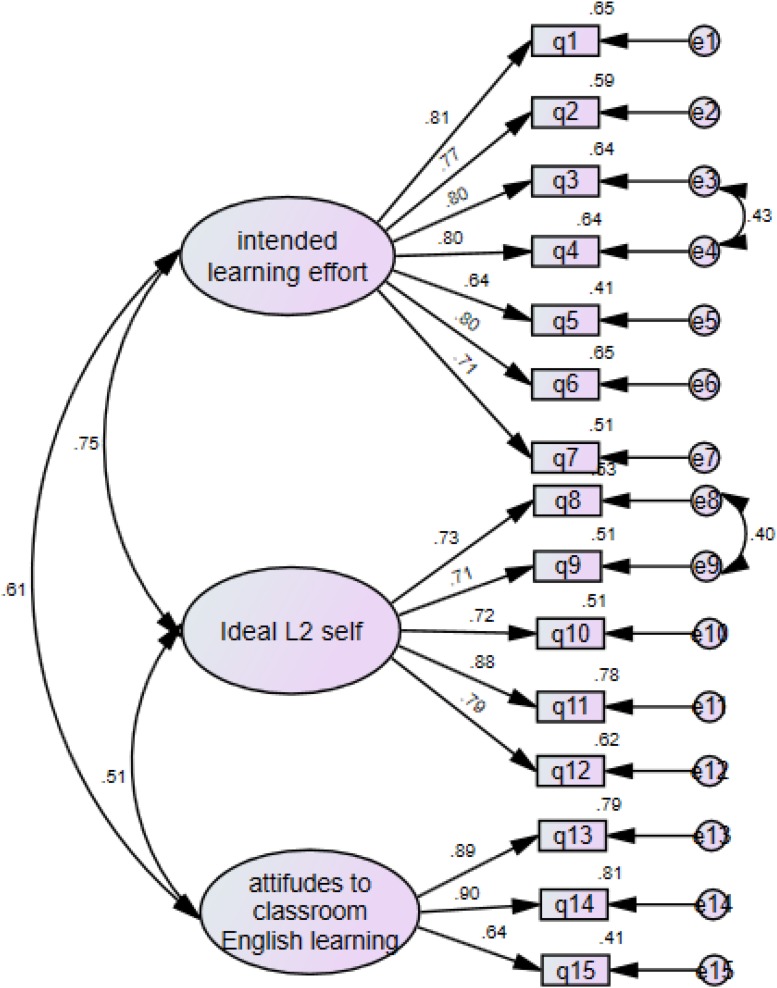
The first-order model of learning motivation.

In addition, CFA was conducted to determine whether the SLMQ responses fit the pre-determined three-factor learning motivation model. Satisfactory model fits were found with χ^2^ = 87.11; df = 85; CFI = 0.95; TLI = 0.95; SRMR = 0.047; RMSEA = 0.076. The Cronbach’s α for the three subscales of this three-factor learning motivation model (Figure 3) were 0.906 (*intended learning effort*, 7 items), 0.882 (*ideal L2 self*, 5 items), and 0.846 (*attitudes to classroom English learning*, 3 items) respectively, indicating very satisfactory levels of internal consistency. These results confirm the three motivational dimensions here as reliable and valid components of second language motivational construct frequently discussed in the literature.

### Chinese University EFL Students’ Feedback Experience and Preference, and English Learning Motivation

Descriptive analysis was conducted to examine profiles of the participants’ feedback experience and feedback preference. As can be seen in Table 1, the mean scores for the two feedback experience scales were 4.108 (q*uantity and quality of feedback*) and 4.097 (*feedback use*), indicating an overall positive endorsement of the quantity and quality of feedback received from lecturers as well as a generally positive engagement with teacher feedback. The mean scores for the three feedback preference subscales were 4.945 (*preference for learning process-oriented feedback*), 4.418 (*preference for* s*tudent self-feedback*), and 4.968 (*preference for teacher evaluative feedback*). Subsequent one-way repeated measures ANOVA analysis showed that there was a significant effect of feedback preference type, *F*(2, 407) = 102.14, *p* < 0.001, partial η^2^ = 0.33 (see Table 2). *Post hoc* comparisons further showed that *preference for self-feedback* (*M* = 4.42, SD = 0.79) was significantly lower than *preference for process-oriented feedback* (*M* = 4.95, SD = 0.90) and *preference for teacher evaluative feedback* (*M* = 4.97, SD = 0.78) respectively.

**TABLE 1:** Descriptive statistics reliability analysis for feedback experience/preference and motivational factors.

	*Mean*	*SD*	Cronbach’s α
Quantity and quality of feedback	4.047	0.944	0.892
Feedback use	4.097	0.911	0.812
Preference for learning process-oriented feedback	4.945	0.900	0.928
Preference for student self-feedback	4.418	0.786	0.834
Preference for teacher evaluative feedback	4.968	0.779	0.818
Intended learning effort	4.713	0.871	0.906
Ideal L2 self	5.010	0.856	0.882
Attitudes to classroom English learning	4.228	1.054	0.846

**TABLE 2:** One-way repeated measures ANOVA results comparing different types of feedback preferences students reported.

	Types		Mean difference	Mean (SD)
Feedback preference	Process oriented feedback	Self feedback	0.527***	4.95 (0.90)
*F*(2, 407) = 102.14,		Teacher evaluative feedback	–0.023	
*p* < 0.001,	Self-feedback	Process-oriented feedback	−0.527***	4.42 (0.79)
partial η^2^ = 0.33		Teacher evaluative feedback	−0.550***	
	Teacher evaluative feedback	Process-oriented feedback	0.023	4.97 (0.78)
		Self-feedback	0.550***	

***p < 0.001.

With regard to motivation, the mean values of the three motivational dimensions range from 5.010 (*ideal L2 self*) to 4.228 (*attitudes to classroom English learning*), revealing a generally favorable disposition toward learning English. The scale value for *intended learning effort* is 4.714, indicating solid commitment. It can thus be concluded that the participants are generally favorably disposed toward studying English as a foreign language.

### Differences in Feedback Experience and Preference Between First-Year, Second-Year, and Third-Year Students

Means, standard deviations, and MANOVA results for feedback experience and preference between first-year, second-year, and third-year students are presented in Table 3. As can be seen from Table 3, there was a statistically significant difference in feedback experience and preference based on students’ year-levels, *F*(10, 788) = 3.11, p = 0.001; Wilk’s Λ = 0.93, partial η^2^ = 0.04. *Post hoc* comparisons indicated that the mean for Year 1 (*M* = 4.31, SD = 0.99) on quantity and quality of teacher feedback was significantly higher than the mean for Year 2 (*M* = 3.88, SD = 0.97). Year-1 was also higher than Year 3 (*M* = 4.09, SD = 0.87) on quantity and quality of teacher feedback, but this difference did not reach significance level. *Post hoc* comparisons also indicated that the mean for Year 1 (*M* = 4.38, SD = 0.92) on feedback use was significantly higher than means for Year 2 (*M* = 4.01, SD = 0.94) and Year 3 (*M* = 3.97, SD = 0.84), respectively.

**TABLE 3:** MANOVA results about feedback experience and preference by university year-level.

	Factors	Year 1 Mean (SD)	Year 2 Mean (SD)	Year 3 Mean (SD)	F	Partial Eta Squared
*F*(10,788) = 3.11,	Quantity and quality of feedback	4.31 (0.99)	3.88 (0.97)	4.09 (0.87)	5.91**	0.029
*p* = 0.001,	Feedback use	4.38 (0.92)	4.01 (0.94)	3.97 (0.84)	9.51***	0.046
Wilk’s Λ = 0.93,	Process-oriented feedback	5.02 (0.88)	4.96 (0.80)	4.91 (0.96)	0.69	0.003
partial η^2^ = 0.04.	Self-feedback	4.53 (0.79)	4.41 (0.69)	4.36 (0.81)	1.98	0.010
	Teacher evaluative feedback	5.03 (0.76)	4.86 (0.85)	4.99 (0.75)	1.27	0.006

**p < 0.01, ***p < 0.001.

### Relationships Between English Learning Motivational Factors and Feedback Experience and Preference

**FIGURE 4:**
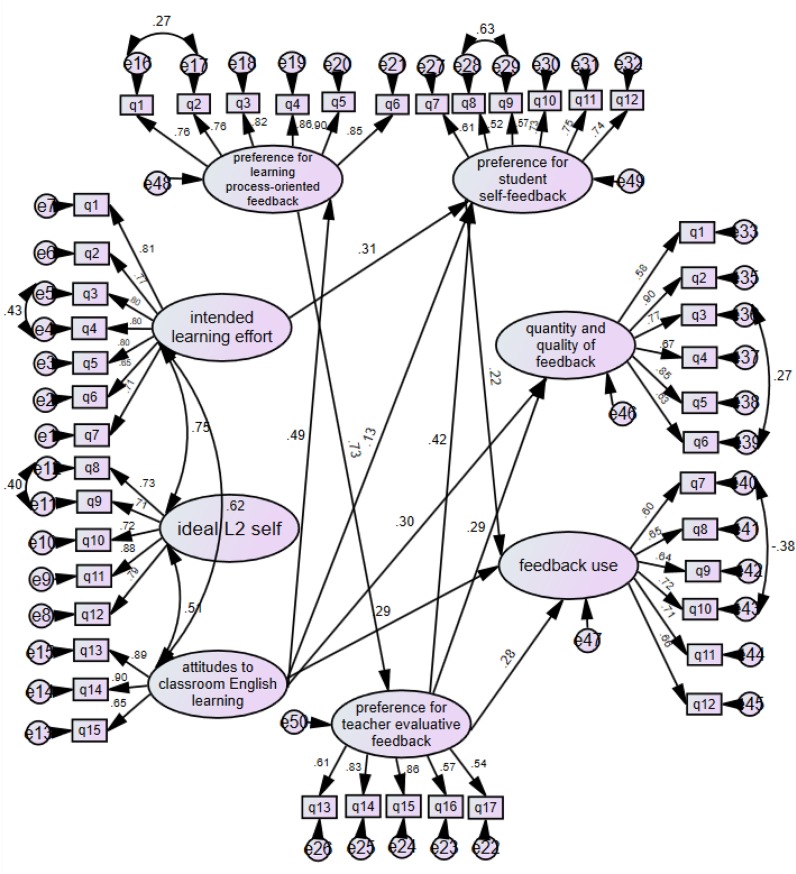
Full structural equation model.

In accordance with the assumption that motivational beliefs as an essential individual factor would influence feedback experience (Nicol and Macfarlane-Dick, 2006), a structural model (Figure 4), with good fit (χ^2^ = 1911.77; df = 921; χ^2^/df = 2.08, CFI = 0.912; TLI = 0.905; SRMR = 0.065; RMSEA = 0.051), revealed some statistically significant paths between second language learning motivational variables and the participants’ feedback preference and experience. Among the three motivational factors, *attitudes to classroom English learning* had significant direct and indirect positive influence on both feedback preference (i.e., *preference* for *learning process-oriented feedback*, and *preference for student self-feedback*) and feedback experience (i.e., *quantity and quality of feedback*, and *feedback use*). Another motivational factor, *intended learning effort*, demonstrated a significant positive influence only on *preference for student self-feedback*, but it further had an indirect positive influence on *feedback use* through *preference for student self-feedback*. The remaining motivational factor, *ideal L2 self*, showed no significant relation with either student feedback preference or feedback experience. Interestingly, between the three feedback preference factors, *preference for learning process-oriented feedback* significantly positively predicted *preference for teacher evaluative feedback*. In addition, *preference for learning process-oriented feedback* had a significant positive indirect influence on *preference for student self-feedback* via *preference for teacher evaluative feedback*.

## Discussion

While feedback is widely acknowledged to be a vital component in successful second language learning, and has been the subject of intensive research in recent years, little has been reported about how EFL students understand and experience different forms of feedback practice within the classroom, and how language motivational factors are associated with students’ feedback experience and preference particularly in an Asian EFL context. This study represents the first attempt to gain insights into Chinese EFL students’ feedback experience and preference, and their associations with EFL learning motivational variables. In the following, the results of this study are discussed with respect to the research questions posed in this study.

### What May Characterize Chinese University EFL Students’ Feedback Experience and Preference, and English Learning Motivation?

The first research question asks what may characterize Chinese university EFL students’ feedback experience and preference, and English learning motivation. Using the EFL SFEQ adapted from Gibbs and Simpson’s (2004) Assessment Experience Questionnaire, we identified two major aspects of Chinese university EFL students’ feedback experience: (1) *quantity and quality of feedback*, and (2) *feedback use*. Cronbach’s alpha coefficients for these two feedback experience factors revealed a robust internal reliability of the scales, suggesting that EFL students make a fine distinction between the feedback itself and what they do with it. The reasonably high mean scores of these two factors indicate that the participants generally found teachers’ feedback helpful, and that they made generally active use of teacher feedback as part of a process of self-regulated learning. These results are generally positive in contrast with persistent student dissatisfaction with feedback processes reported in studies in general education (Carless and Boud, 2018).

Using the EFL SFPQ developed for this study, we identified three major types of feedback preference in Chinese university EFL students: (1) *Preference for learning process-oriented feedback*; (2) *Preference for student self-feedback*; (3) *Preference for teacher evaluative feedback*. The students demonstrated a greater preference for teacher-led feedback practices than for student self-feedback. Previous studies with students studying in a student-centered curriculum environment with a strong commitment to formative learning practices in the western society also found that students tend to dominantly select and endorse teacher-led feedback practices (Harris et al., 2014). It might thus be natural for students to generally pay more attention to teacher-led feedback than self- and/or peer-feedback probably as a consequence of the promise of teacher feedback giving directions for future improved learning (Nicol and Macfarlane-Dick, 2006). This may particularly be the case in the EFL context where assisted by a more knowledgeable other (i.e., a teacher), students can experience models of successful language learning and participate in more complex social activities (Lave and Wenger, 1991). On the other hand, however, a mean score of 4.039 on *preference for student self-feedback* in this study is also a moderately favorable result and is one that EFL teachers should look to exploit as this suggests that students might have already developed an awareness that feedback can also come legitimately from themselves and their peers. Given the ongoing debate on whether student autonomous learning practices can be established in the Confucian learning culture, this finding is encouraging and provides support to the case for the use of feedback to empower students as self-regulated learners.

Using the EFL Student Learning Motivation Questionnaire that was developed specifically for this study based on some key motivational constructs from Dörnyei (2005, 2009) L2 Motivational Self System and the related research (e.g., Csizér and Kormos, 2009; Taguchi et al., 2009), a three-dimensional structure of Chinese university EFL students’ English learning motivation has been identified, lending support to application of some key constructs of Dörnyei’s motivational theory to the Chinese EFL learning settings: (1) *intended learning effort*; (2) *ideal L2 self*, and (3) *attitudes to classroom English learning*. The higher mean scores on *ideal L2 self* and *intended learning effort* versus lower mean scores on *attitudes to classroom English learning* imply a possibly more positive endorsement of individuals’ future desired selves and individuals’ intended efforts. This result appears to be congruent with Taguchi et al. (2009) observation that compared with future desired selves, classroom EFL learning experience is less important for Chinese students.

### Are There Differences in Feedback Experience and Preference Between First-Year, Second-Year, and Third-Year Students?

The second research question asks whether there are differences in feedback experience and preference between first-year, second-year, and third-year students. This study found that lower year-level students appeared to have greater preference for and use of teacher feedback than higher year-level students. It might be that higher year-level students were likely to take a more critical approach to feedback, and might be more self-reliant in their EFL learning as they progress through higher education. Nevertheless, there might be other reasons why higher year-level students demonstrated a generally lower level of preference for and use of teacher feedback as Gibbs and Simpson (2004) noticed that third-year university students were particularly likely only to look at the grade rather than at feedback on essays. More research is undoubtedly needed to explore the potential variation in feedback preference and use across different year-levels.

### What Are the Relationships Between English Learning Motivational Factors and These EFL Students’ Feedback Experience and Preference?

The third research question inquires into the relationships between English learning motivational factors and EFL students’ feedback experience and preference. This study is the first of its kind to examine the associations between some major constituents of Dörnyei’s L2 Motivational Self System and students’ feedback processes in the EFL context. In this study, structural equation modeling (SEM) was conducted to examine how motivational variables were related to EFL students’ feedback experience and preference. Students’ *attitudes to classroom English learning* was found to play the most important role in influencing their classroom feedback experiences and preferences. This result suggests that students who are actively interested in the process of classroom English learning are more likely to assume an agentic role in feedback processes. The result of *attitudes to classroom English learning* having the highest impact in the present study confirms the finding obtained in previous research (Csizér and Kormos, 2009; Papi, 2010). Meanwhile, it is also interesting to note that *ideal L2 self* had no significant direct or indirect impact on any feedback experience or preference. These two results can be interpreted as suggesting that, although the students tend to highly envision selves being proficient in English in the future, such envisioning may exist independently of their current classroom English teaching and learning reality which is usually examination-oriented and may bring about some adverse effect on students’ attitudes toward learning, and which presumably results in their inability “to afford the luxury of caring for the niceties of the classroom experience” (Taguchi et al., 2009, p. 87). Therefore, the *ideal L2 self* may not play a substantial role in determining student feedback experience and preference, which is essentially a rather situation-specific factor related to the students’ immediate L2 learning environment (Dörnyei, 2009). Similarly, the significant effect of *intended learning effort* on *preference for student self-feedback* is also congruent with Kormos and Csizér’s (2014) finding that a willingness to invest effort into language learning in general acts as an important driving force in students’ readiness to actively seek opportunities for learning. Furthermore, in this study, the role that *intended learning effort* plays is two-dimensional as it also contributes to feedback use indirectly, via impacting *preference for student self-feedback*.

The SEM analysis also revealed insights into the interesting and complex interrelationships between feedback preference (i.e., *preference for learning process-oriented feedback, preference for teacher evaluative feedback*, and *preference for student self-feedback*) and feedback experience (i.e., *perceived quantity and quality of feedback*, and *feedback use*) in this study. It is suggested in the literature that students who hold *mastery-learning* goals embrace feedback aimed at improving learning strategies and processes, whereas students who hold performance goals embrace feedback about how well a task is being accomplished or performed, i.e., corrective or evaluative feedback. Leenknecht et al. (2019) also reported a high correlation between mastery goal orientation and performance goal orientation. This means that students with mastery goals may also embrace performance goal orientation. In other words, it is likely that students who embrace learning strategies- or processes-oriented feedback may also embrace corrective or evaluative feedback. This result which is congruent with recent feedback engagement research (e.g., Han, 2017) in SLA appeared to be further supported by the SEM analysis result that *preference for learning process-oriented feedback* significantly predicted *preference for teacher evaluative feedback*. But the vice-versa was not true with our study participants. Furthermore, *preference for teacher evaluative feedback* significantly predicted *feedback use* and *quantity and quality of feedback* as well as *preference for student self-feedback*, whereas *preference for learning process-oriented feedback* revealed no direct influence on those three feedback variables. These results suggest that feedback for teacher evaluative feedback played the dominant role in the university feedback culture. The result that *preference for learning process-oriented feedback* influenced other feedback variables indirectly via *preference for teacher evaluative feedback* further suggested that within this type of university feedback culture, the power of learning process-oriented feedback could be diluted by teacher evaluative feedback. This study thus provides evidence that while students reported a good level of preference for learning process-oriented feedback, this preference may not convert into action. The dominant role of *preference for teacher evaluative feedback* could be attributed to an examination-oriented culture where testing is used as the measure to evaluate whether change in student performance has occurred rather than as a mechanism to further enhance learning by teachers or students (Hattie and Timperley, 2007; Carless, 2011). Hattie and Timperley (2007) argued that within the classroom context, the most beneficial feedback was process-oriented feedback as it provides students with opportunities to be proactive in the feedback process and to exert agency in their learning (Sadler, 1989; Hawe et al., 2008). Given our findings, however, students’ preference for teacher evaluative feedback could somewhat constrain students’ action on learning process-oriented feedback in the Chinese EFL learning context. Consequently, to contemplate and plan how learning process-oriented feedback can be effected, interventions could be designed and implemented to raise both teacher’s and students’ awareness that feedback needs to be understood or used in ways that contribute to *both* learning achievement *and* improvement of learning strategies and processes that lead to a deep understanding of learning.

While this study adds to knowledge pertaining to the dynamic relationships between EFL motivational factors and students’ feedback experience and preference, a number of limitations need to be acknowledged. First, this study relied on the use of self-reported data, which might have resulted in the common method variance, a situation that might have inflated the true associations between variables (Teo et al., 2017). Future observational qualitative classroom-based research is needed to complement the statistical evidence reported here so as to provide a more detailed understanding of the associations between motivational factors and students’ feedback experience. Secondly, although the participants in this study were from four different mainland Chinese universities, they were all English-major students, and consequently, the results of this study may not be well representative of students in Chinese higher education. Including students from a wide range of academic subject areas would help future research to gain deeper insights into the impact of language motivational impact on EFL students’ feedback experience and preference in the Asian settings.

## Conclusion

This study contributes to knowledge about how students experience feedback in an EFL classroom context. The results of the study add to the literature that considers influence of individual factors on learner feedback engagement, and suggest that different components of the second language learning motivational construct appear to display differential impacts on EFL students’ feedback experience and preference. In particular, the study brought to light the crucial role of *attitudes to classroom English learning* and *intended learning effort* as essential mediating motivational variables in predicting how EFL students conceive of and act on feedback. The findings of this study point to the conclusion that Chinese EFL students’ feedback preferences and involvement in feedback processes are mainly mediated by their attitudes toward the immediate learning environment/experience and their intended learning efforts. This study also revealed that different constructs from Dörnyei’s L2 Motivational Self System have shown different levels of predictive relationships with feedback engagement/preference variables, with *ideal L2 self* being the weakest. Further research is needed to understand how other motivational constructs such as goal orientation and language mindsets (Dweck and Leggett, 1988; Lou and Noels, 2019) may impact student feedback engagement and learning outcomes. Pedagogically, since teachers exert an important influence on what attitudes students have toward classroom learning experience, we therefore argue that EFL teachers need to shoulder the burden of making the EFL classroom a supportive environment that promotes a positive self-concept and self-confidence as the first step toward minimizing potential negative emotional well-being among students and stimulating students’ active feedback use.

The findings of this study also provide significant insights into a complex and dynamic view of how student preference for different types of feedback actually works in the feedback process. The study participants reported a generally high level of preference for learning process-oriented feedback, but there was sign that this type of feedback was not usually acted upon. The pedagogical implication of the findings seems straightforward. Since learning process-oriented feedback consistently pitched at the deep learning level will allow the learner to progress toward achieving more complex learning goals (Harris et al., 2015), we expect that conditions need to be created to allow for connection of students’ preference for process-oriented feedback to action to maximize the pivotal role that students play in the classroom and learning process. While the potential discrepancy between preference for process-oriented feedback and its conversion to action among Chinese university EFL students documented in this study is undoubtedly worth further investigation, professional development is needed to equip EFL teachers with theory about and experience of high-quality feedback to raise their awareness of the feedback practices that involve students in giving themselves and each other feedback that supports a deep understanding of learning.

In this study, we aimed to establish an adequate and empirically supported model of second language learning motivation, feedback preference, and feedback experience, in a Chinese EFL learning context. The findings of our study may be applicable to other settings where English is taught and learned as a foreign language, and where teachers tend to play a dominant role in the classroom. However, as motivational factors and feedback behavior show contextual variations, further research in other social and educational contexts is needed to examine how the interaction of the variables investigated might be influenced by social-contextual factors (Kormos and Csizér, 2014).

## Data Availability Statement

The datasets generated for this study are available on request to the corresponding author.

## Ethics Statement

The studies involving human participants were reviewed and approved by The Research Ethics Committee of the University of Macau. The patients/participants provided their written informed consent to participate in this study.

## Author Contributions

The author confirms being the sole contributor of this work and has approved it for publication.

## Conflict of Interest

The authors declares that the research was conducted in the absence of any commercial or financial relationships that could be construed as a potential conflict of interest.
